# Relative Validity of Starch and Sugar Intake in Japanese Adults as Estimated With Comprehensive and Brief Self-Administered Diet History Questionnaires

**DOI:** 10.2188/jea.JE20190026

**Published:** 2020-08-05

**Authors:** Aya Fujiwara, Kentaro Murakami, Satoshi Sasaki

**Affiliations:** 1 Department of Social and Preventive Epidemiology, Graduate School of Medicine, The University of Tokyo, Tokyo, Japan; 2 Department of Social and Preventive Epidemiology, School of Public Health, The University of Tokyo, Tokyo, Japan

**Keywords:** relative validity, starch and sugars, diet history questionnaire, Japanese

## Abstract

**Background:**

In Japan, large-scale epidemiological studies on starch and sugar intake are scarce, mainly due to a lack of a suitable assessment tool. We examined the relative validity of two widely-used dietary assessment questionnaires for Japanese adults, the comprehensive Diet History Questionnaire (DHQ) and the brief DHQ (BDHQ), for estimating the intake of starch and 10 types of sugars: total sugar, sucrose, maltose, lactose, trehalose, glucose, fructose, galactose, and added and free sugars.

**Methods:**

A total of 92 women and 92 men completed 4-day weighed dietary records (DRs) besides the DHQ and BDHQ in each of the four seasons. For each method, starch and sugar intake was calculated according to a recently developed food composition database on starch and sugars for Japanese food items.

**Results:**

For most of the carbohydrate variables examined, the median energy-adjusted intake derived from the first DHQ and BDHQ (DHQ1 and BDHQ1, respectively) significantly differed from those derived from the 16-day DRs in both sexes. Spearman correlation coefficients between the 16-day DRs and DHQ1 were acceptable (≥0.31) for all variables (0.31–0.67), except for maltose and trehalose in women (≤0.29). For BDHQ1, the correlations were also acceptable for all variables (0.32–0.64), except for maltose (≤0.26) and galactose (≤0.06). Similar results were observed for the mean of four DHQs and BDHQs.

**Conclusions:**

This study indicated a reasonable ranking ability of DHQ and BDHQ for the intake of starch and most sugars examined, despite a poor ability to estimate the intake at the both group and individual levels.

## INTRODUCTION

Dietary carbohydrates have attracted considerable attention due to their potential influence on human health,^1^ particularly dental caries,^2^ overweight,^3^ type 2 diabetes,^4^ and cardiovascular diseases.^5^^,^^6^ The effects on health possibly differ between carbohydrate subtypes, such as starch, total sugar, and saccharides, as well as naturally occurring and added sugars.^7^^–^^11^ To investigate their health effects, it is essential to develop an assessment method for each of these carbohydrates.

Dietary assessment questionnaires are widely used in large-scale epidemiological studies because of their feasibility and low cost.^12^ In Western countries, many studies have reported the validity of these questionnaires for the intake of carbohydrate subtypes, such as starch,^13^^–^^20^ total sugar,^13^^–^^17^^,^^19^^–^^22^ and sucrose,^15^^,^^17^^,^^18^^,^^23^^–^^26^ although studies on the validity of intake of added sugar^27^ and saccharides, including lactose,^15^^,^^17^^,^^24^ glucose,^15^ fructose,^15^^,^^17^ and galactose,^15^ are limited. Conversely, there are only a few Asian studies,^28^^,^^29^ mainly due to the lack of a comprehensive composition database on starch and sugars in Asian countries, including Japan. However, Fujiwara et al^30^^,^^31^ recently developed a comprehensive database on the starch and sugar content of foods commonly consumed in Japan based on the Standard Tables of Food Composition in Japan (STFCJ)^32^^,^^33^ and estimated the intake of these carbohydrates in a Japanese population with a wide age rage using dietary records (DRs). The mean intake of sugars in the Japanese population was lower than that of Western countries, whereas the starch intake was comparable.^30^^,^^31^ Thus, the relationship between sugar intake and health status in the Japanese population could differ from that in the Western population. To investigate the relationship between starch and sugar intake and health status, a feasible dietary assessment tool is needed for the Japanese population, such as a questionnaire.

Dietary assessment questionnaires like the comprehensive self-administered diet-history questionnaire (DHQ) and a brief self-administered DHQ (BDHQ) for Japanese adults^34^^–^^40^ assess dietary intake during the preceding month and are widely used in Japan. Here, we examined the relative validity of the intake of starch and 10 types of sugars (total sugar, sucrose, maltose, lactose, trehalose, glucose, fructose, galactose, and added and free sugars) as estimated using the DHQ and BDHQ, with 16-day semi-weighed DRs as the reference.

## METHODS

### Study design

Details of the study design, participant characteristics, and dietary assessment methods have been previously reported.^38^ Briefly, the present study was conducted in three areas of Japan—Osaka (urban), Nagano (rural inland), and Tottori (rural coastal)—from November 2002 to September 2003. In each area, apparently healthy women aged 30 to 69 years who were willing to participate with a cohabitating husband were recruited, such that each of the four 10-year age class strata had eight women, regardless of the age of the husbands. Thus, a total of 96 women and 96 men were recruited.

The survey purpose and protocol were explained before the survey, and written informed consent was obtained from each participant. The present study did not require ethics approval because it was conducted before the advent of ethics guidelines for epidemiology research in Japan. However, the use of the data from the study was approved by the Ethics Committee at the University of Tokyo Faculty of Medicine (No. 3421).

The dietary assessment was conducted in each of the four seasons at intervals of approximately 3 months, namely in November and December 2002 for fall, February 2003 for winter, May 2003 for spring, and August and September 2003 for summer, using 4-non-consecutive-day semi-weighed DRs (ie, 16-day DRs in total). For the DRs, each of the four recording days consisted of 3 weekdays and 1 weekend day within approximately 2 weeks. The DHQ and BDHQ were answered approximately 2 days before the start of the DRs in each season, in the order of BDHQ before DHQ. In the present study, the data from the first DHQ and BDHQ (DHQ1 and BDHQ1, respectively) were used to examine whether a single DHQ and BDHQ could represent habitual dietary intake over a longer period (eg, 1 year) although the study period differed between the first DHQ or BDHQ (previous month in autumn) and the 16-day DRs (four seasons from autumn to summer). This is because many epidemiological studies administer dietary assessment questionnaires only once. The data from the DHQ1 and BDHQ1 were used for this purpose because the answers provided from other DHQ and BDHQ (conducted after the experience of answering the DRs) may have been influenced by the attention to diet required to complete the DRs. Additionally, the mean values of the four DHQs (mDHQ) and BDHQs (mBDHQ) (ie, covering four seasons) were examined in the same manner to match the reference period with that of the 16-day DRs. In total, all 92 women and 92 men completed the protocol and were included in the present analysis.

### Dietary records

Participants were given both written and verbal instructions on how to keep the DRs and were provided with recording sheets and a KD-173 digital scale (Tanita, Tokyo, Japan; precision ±2 g at 0–250 g and ±4 g at 251–1,000 g). They were asked to weigh and record all foods and beverages consumed during the survey period. When they encountered difficulties in weighing (eg, dining out), they were asked to document as much information as possible, including the brand name of the food, the consumed portion size (based on typical household measures), as well as the details of leftovers. The participants were instructed to fax the completed recording sheets for each recording day to the local staff. The local staff then reviewed the record and, whenever necessary, collected additional information for the record via telephone or fax. The responses were generally faxed to the centre, although some were handed directly to the centre staff. All the DRs were collected and checked by trained registered dietitians in the local centres and then again in the study centre. As requested in the study protocol, portion sizes estimated using household measures were converted into weights, and individual food items were coded based on the STFCJ.^43^ A total of 1,299 food and beverage items appeared in the DRs.

### DHQ and BDHQ

Details of the structure, calculation method, and validity of the DHQ and BDHQ have been described elsewhere.^34^^–^^40^ Briefly, the DHQ is a 16-page semi-quantitative questionnaire that assesses the consumption frequency and portion size of selected foods to estimate the dietary intake of 150 food and beverage items during the preceding month. The DHQ consists of seven sections: (1) general dietary behaviour; (2) usual cooking methods; (3) consumption frequency and amount of alcoholic beverages; (4) consumption frequency and semi-quantitative portion size of selected food and non-alcoholic beverage items; (5) dietary supplements; (6) consumption frequency and semi-quantitative portion size of staple foods, soup for noodles, and miso (fermented soybean paste) soup; and (7) open-ended items for foods consumed more than once a week but not appearing in the DHQ. The BDHQ is a 4-page fixed-portion questionnaire that assesses the consumption frequency of selected foods, but not portion size, to estimate the dietary intake of 58 food and beverage items during the preceding month.^39^^,^^40^ The BDHQ consists of five sections: (1) intake frequency of food and non-alcoholic beverage items; (2) daily intake of rice and miso soup; (3) frequency of drinking alcohol and amount of alcohol per drink for alcoholic beverages; (4) usual cooking methods; and (5) general dietary behaviour. Estimates of the intake of food and beverage items listed in the DHQ and BDHQ were calculated using an *ad hoc* computer algorithm for each dietary assessment questionnaire.^34^^–^^40^

The relative validity of the intake of carbohydrates as well as food groups using a single DHQ and BDHQ was previously assessed using 16-day DRs in the present population.^39^^,^^40^ Pearson correlation coefficients for carbohydrate between the 16-day DRs and the DHQ1 were 0.58 in women and 0.67 in men, respectively, and the corresponding values of the BDHQ1 were 0.48 and 0.64.^39^ For food groups, median Spearman correlation coefficients between the 16-day DRs and the DHQ1 were 0.43 (range: −0.09–0.77) for women and 0.44 (range: 0.08–0.87) in men, and the corresponding values of the BDHQ1 were 0.44 (range: 0.14–0.82) and 0.48 (range: 0.22–0.83).^40^

Responses to the DHQ and BDHQ were checked at least twice for completeness by dietitians. When missing answers or logical errors were identified, the participants were asked to complete the questions again.

### Calculation of intake of energy, starch, and 10 types of sugars

Based on the food intake estimated using the mean of 16-day DRs as well as the DHQ and BDHQ, daily energy intake (kcal/d) was calculated according to the STFCJ.^43^ Similarly, the daily intake (g/d) of starch and 10 types of sugars (total sugar, sucrose, maltose, lactose, trehalose, glucose, fructose, galactose, and added and free sugars) were calculated according to the starch and sugar composition database for the Japanese.^30^ For the DHQ, some items include multiple similar foods. For example, green leafy vegetables in the DHQ consist of spinach mustard, leaves of Japanese radishes, green bok choy, leaves of Welsh onions, and spinach. Consequently, the DHQ algorithm consists of 211 items in the STFCJ^41^ and the starch and sugar composition database. Similarly, some items in the BDHQ include multiple similar foods and thus, the BDHQ algorithm consists of 147 items in these databases. Total sugar was defined as the sum of all mono- and disaccharides, including glucose, fructose, galactose, sucrose, lactose, maltose, and trehalose.^42^ Free sugar was defined as all monosaccharides and disaccharides added to foods by the manufacturer, cook or consumer, plus sugars naturally present in honey, syrups, and fruit juices.^43^ As added sugar was defined as sugars and syrups added to food during processing or preparation, excluding naturally occurring sugars in food,^44^ added sugar contents in food items were calculated as subtracting total sugar contents derived from fruit juices from free sugar contents.

### Statistical analysis

All the statistical analyses were conducted for women and men separately using SAS statistical software version 9.4 (SAS Institute Inc., Cary, NC, USA). The relative validity was examined using energy-adjusted values via the residual (g/d) and density (percent of energy intake) methods to account for misreporting of dietary intake.^12^^,^^45^ For the residual method, the energy-adjusted intake (g/d) of each participant was calculated as the sum of the mean intake of each nutrient (or food) in the study population and the residual for each participant from the regression model, which included the nutrient (or food) intake of the study population as the dependent variable and the energy intake of the study population as the independent variable.^12^ The Shapiro-Wilk test for normality suggested that 71.4% of energy-adjusted intake of starch and each type of sugar estimated using the DRs, DHQ, and BDHQ were non-normally distributed. Therefore, the non-parametric test was used in the present study. Median and interquartile range (IQR) values for intake of starch and each type of sugar were calculated for the mean of the 16-day DRs, DHQ1, and BDHQ1 as well as for the mDHQ and mBDHQ. To assess the ability to estimate group median intake, differences in the median intake between the median of the 16-day DRs and the DHQ1, BDHQ1, mDHQ, and mBDHQ were analysed using a Wilcoxon signed-rank test. A *P*-value less than 0.05 was considered to indicate significance. Moreover, Spearman correlation coefficients between the intakes of the 16-day DRs and the DHQ1, BDHQ1, mDHQ and mBDHQ were calculated to assess the ranking ability for individual intake. Because a previous review of validation studies of dietary questionnaires developed and validated in Japan reported that medians of correlation coefficients between DRs and questionnaires ranged from 0.31 to 0.56,^46^ the correlation coefficients of ≥0.31 were defined as acceptable. Bland-Altman plots were undertaken to investigate the agreement in individual intake between the mean of the 16-day DRs and the DHQ1, BDHQ1, mDHQ, and mBDHQ.^47^^,^^48^

The crude mean intake of starch and each type of sugar from each food group from the 16-day DRs, as well as from the DHQ1 and BDHQ1, were calculated to clarify the major food sources. Food items were grouped based on similarities in nutrient profile or culinary use of the foods, mainly in accordance with the STFCJ^41^ and a previous study^40^ (Table 1).

**Table 1.** Definition of food groups

Food groups^b^	Food items^a^	

	DHQ	BDHQ
Rice	Well-milled rice; well-milled rice mixed with barley; well-milled rice with germ; half-milled rice; 70% milled rice; brown rice (*n* = 6)	Rice (*n* = 1)
Bread	White bread; rolled bun; croissant; pizza; Japanese-style pancakes; sweet bun with a sweet filling, such as azuki bean paste, custard cream, and jam; pancakes (*n* = 7)	Breads (including white bread and sweet bun with a sweet filling, such as azuki bean paste, custard cream, and jam) (*n* = 1)
Noodles	Japanese noodles (buckwheat and Japanese wheat noodles); instant noodles; Chinese noodles; spaghetti (*n* = 4)	Buckwheat noodles; Japanese wheat noodles; instant noodles and Chinese noodles; spaghetti and macaroni (*n* = 4)
Other grain products^c^	Cornflakes (*n* = 1)	NA
Potatoes	French fries; potatoes; sweet potatoes, yams and taro; konnyaku (ie, devil’s tongue jelly) (*n* = 4)	Potatoes (all varieties) (*n* = 1)
Sugars	Jam and marmalade; sugar for coffee and black tea; sugar used during cooking (*n* = 3)	Sugar for coffee and black tea; sugar used during cooking (*n* = 2)
Pulses	Tofu (ie, soyabean curd); tofu products; natto (ie, fermented soyabeans); boiled beans; (*n* = 4)	Tofu (ie, soyabean curd) and tofu products; natto (ie, fermented soyabeans) (*n* = 2)
Nuts^c^	Peanuts; other nuts (*n* = 2)	NA
Vegetables	Carrots; pumpkins; tomatoes; green peppers; broccoli; green leafy vegetables; cabbage; cucumbers; lettuce; Chinese cabbage; bean sprouts; radishes; onions; cauliflower; eggplants; burdock; lotus root; salted pickled plums; other salted pickles (*n* = 19)	Carrots and pumpkins; tomatoes, tomato ketchup, boiled tomato and stewed tomato; green leafy vegetables including broccoli; raw vegetables used in salad (cabbage and lettuce); cabbage and Chinese cabbage; radishes and turnips; other root vegetables (onions, burdock and lotus root; salted green and yellow vegetable pickles; other salted vegetable pickles (excluding salted pickled plum) (*n* = 9)
Mushrooms	Mushrooms (*n* = 1)	Mushrooms (all varieties) (*n* = 1)
Seaweeds	Wakame and hijiki seaweed; laver (ie, dried, edible seaweed) (*n* = 2)	Seaweeds (all varieties) (*n* = 1)
Fruits	Raisins; canned fruit; oranges; bananas; apples; strawberries; grapes; peaches; pears; persimmons; kiwi fruit; melons; watermelons (*n* = 13)	Citrus fruit including oranges; strawberries, persimmons and kiwi fruit; other fruits (*n* = 3)
Fish and shellfish	Dried fish; small fish with bones; canned tuna; eel; white meat fish; oily fish; red meat fish; ground fish meat products; shrimp and crab; squid and octopus; oysters; other shellfish; fish eggs; boiled fish and shellfish in soya sauce; salted fish intestines (*n* = 15)	Dried fish and salted fish (including salted mackerel, salted salmon and dried horse mackerel); small fish with bones; canned tuna; oily fish (including sardines, mackerel, saury, amberjack, herring, eel and fatty tuna); non-oily fish (including salmon, trout, white meat fish, freshwater fish and bonito); squid, octopus, shrimp and clam (*n* = 6)
Meat	Ground beef and pork; chicken; pork; beef; liver; ham and sausages; bacon (*n* = 7)	Chicken (including ground chicken); pork and beef (including ground pork and beef); liver; ham, sausages and bacon (*n* = 4)
Eggs	Eggs (*n* = 1)	Eggs (*n* = 1)
Milk	Full-fat milk; low-fat milk; skimmed milk (*n* = 3)	Full-fat milk and yoghurt; low-fat milk and yoghurt (*n* = 2)
Yogurt and cheese^c^	Sweetened yoghurt; non-sweetened yoghurt; moderately sweetened yoghurt; cheese; cottage cheese; cream or creamer added to coffee (*n* = 5)	NA
Fat and oils	Butter; margarine; mayonnaise; salad dressing; oil used during cooking (*n* = 5)	Mayonnaise and salad dressing; oil used during cooking (*n* = 2)
Confectionaries	Chocolates; candies, caramels and chewing gum; potato chips; rice crackers; snacks made from wheat flour; Japanese sweets with azuki beans; Japanese sweets without azuki beans; cakes; cookies and biscuits; jellies; doughnuts; ice cream (regular); ice cream (premium); ice cream (unspecified varieties); nutritional supplement bars (*n* = 15)	Rice crackers, rice cakes and Japanese-style pancakes; Japanese sweets; cakes, cookies and biscuits; ice cream (*n* = 4)
Tea and coffee	Green, barley and oolong tea (including other Chinese tea); black tea; coffee (*n* = 3)	Green tea; black and oolong tea (including other Chinese tea); coffee (*n* = 3)
Sugar sweetened beverages	Lactic acid bacteria beverages; fruit juice excluding 100% juice; cocoa; cola and sugar-sweetened soft drinks (including sports drinks); nutritional supplement drinks (*n* = 5)	Cola and sweetened soft drinks (including sports drinks) (*n* = 1)
Fruit and vegetable juices	Fruit juice (100%); tomato juice; vegetable juice (*n* = 3)	Fruit juice and vegetable juice (100%) (*n* = 1)
Alcohol beverages	Beer; sake; shochu; shochu mixed with water or a carbonated beverage; whiskey; wine (*n* = 6)	Beer; sake; shochu and shochu mixed with water or a carbonated beverage; whiskey; wine (*n* = 5)
Salt	Table salt; salt from noodle soup; salt used during cooking (*n* = 3)	Salt from salt-containing seasonings used at the table such as soy sauce; salt from noodle soup; salt used during cooking (*n* = 3)
Soy sauce^c^	Soy sauce as seasoning (*n* = 1)	NA
Miso	Miso as seasoning; miso for miso soup (*n* = 2)	Miso for miso soup (*n* = 1)
Mirin^c,d^	NA	NA
Other seasonings^c^	Tomato ketchup; non-oil dressing; curry or stew roux; corn soup; Chinese soup (*n* = 5)	NA

DHQ, self-administered diet history questionnaire (150 items); BDHQ, brief self-administered DHQ (58 items); NA, not assessed.

^a^The following food items included in the DHQ (four items) were not categorized: artificial sweeteners; sugar-free soft drinks and diet cola; water for miso soup; and drinking water.

^b^Twenty-eight food groups were defined based on the culinary usage and the similarity of nutrient profiles of the foods, mainly according to the Standard Tables of Food composition in Japan.^41^

^c^The respective food group was not assessed in the DHQ or BDHQ.

^d^Japanese traditional sweet seasoning made from rice, rice koji and distilled alcohol.

## RESULTS

This analysis included 92 women aged 31 to 69 years, with a mean age of 49.6 years, and 92 men aged 32 to 76 years, with mean age of 52.8 years (Table 2). Table 3 shows the results of energy-adjusted intake for both the residual and density methods in the 16-day DRs, DHQ1, and BDHQ1. We described only the results derived from density method in this text because results derived from residual method were similar. In both sexes, the DHQ1 on average overestimated the intake of sucrose, added sugar, and free sugar (median of differences: 0.6–1.5% of energy [%E]) but underestimated intake of maltose and glucose (0.08–0.4%E) compared with the DRs. Additionally, the DHQ1 overestimated total sugar intake in women (0.9%E) but underestimated intake of lactose, trehalose, and fructose in men (0.006–0.1%E). For the BDHQ1, starch intake was overestimated in both sexes (3.9%E in women and 1.8%E in men), whereas the intake of maltose, trehalose, glucose, galactose, added sugar, and free sugar were underestimated (0.009–1.8%E). Furthermore, the BDHQ1 underestimated intake of total sugar (1.4%E) and fructose (0.2%E) in women. Similar findings were observed in mDHQ and mBDHQ (eTable 1).

**Table 2.** Basic characteristic of Japanese women and men

	Women (*n* = 92)		Men (*n* = 92)	

	Mean	SD	Mean	SD
Age (years)	49.6	11.4	52.8	12.1
Height (cm)^a^	155.6	5.8	168.0	6.7
Weight (kg)^a^	53.4	7.1	66.2	11.2
BMI (kg/m^2^)^b^	22.1	2.6	23.3	3.1
Energy intake (kcal/d)				
16-day DRs	1,872	274	2,385	427
DHQ1	1,911	368	2,292	575
BDHQ1	1,760	417	2,208	584
mDHQ	1,878	331	2,337	490
mBDHQ	1,706	334	2,211	485

BDHQ1, first brief self-administered diet history questionnaire; BMI, body mass index; DRs, dietary records; DHQ1, first self-administered diet history questionnaire; mBDHQ, mean of four brief self-administered diet history questionnaires; mDHQ, mean of four self-administered diet history questionnaires; SD, standard deviation.

^a^Measured to the nearest 0.1 cm (height) and 0.1 kg (weight) while the participants wearing light clothing and no shoes.

^b^Calculated by dividing body weight in kilograms by the squared body height in meters.

**Table 3.** Intake of starch and 10 types of sugars estimated using the 16-day DRs, DHQ1, and BDHQ1 among Japanese women and men

	Residual model (g/d)^a^								Density model (% of energy)^a^							

	16-day DRs		DHQ1^b^			BDHQ1^b^			16-day DRs		DHQ1^b^			BDHQ1^b^		

	Median	IQR	Median		IQR	Median		IQR	Median	IQR	Median		IQR	Median		IQR
Women (*n* = 92)																
Starch	153.3	142.8–163.8	154.8		142.1–170.4	160.7		163.7–233.6	32.9	30.6–35.2	33.6		28.8–37.5	36.3	^**^	31.2–40.4
Total sugar	68.3	56.1–76.2	71.1	^**^	62.2–80.5	57.2	^***^	48–70.4	14.5	11.7–16.2	15.1	^**^	12.5–17.1	12.5	^*^	10.7–14.8
Sucrose	31.1	25.7–37.7	38.3	^***^	32.8–44.3	27.6	^***^	22–34.3	6.5	5.3–7.9	8.0	^***^	6.4–9.2	6.2		4.8–7.4
Maltose	1.9	1.4–2.4	1.5	^***^	1.0–2.0	0.8	^***^	0.6–1.3	0.4	0.3–0.5	0.3	^***^	0.2–0.4	0.2	^***^	0.1–0.3
Lactose	7.0	4.7–9.6	7.1		5.3–11.2	6.6		2.4–8.4	1.5	0.9–2.1	1.6		1.0–2.3	1.5		1.1–2.1
Trehalose	0.17	0.11–0.23	0.15		0.10–0.21	0.12	^***^	0.09–0.20	0.04	0.02–0.05	0.03		0.02–0.04	0.03	^***^	0.02–0.04
Glucose	12.5	10.6–14.8	10.6	^***^	9.1–13.6	10.3	^***^	9.3–15.1	2.7	2.3–3.2	2.2	^***^	1.9–2.8	2.3	^***^	1.9–2.8
Fructose	11.2	9.2–13.9	10.7		8.4–14.4	9.8	^***^	7.9–14.2	2.4	2.0–2.9	2.3		1.6–3.0	2.2	^*^	1.7–2.8
Galactose	0.27	0.12–0.54	0.24		0.14–0.63	0.03	^***^	0.02–0.05	0.06	0.03–0.11	0.05		0.02–0.13	0.006	^***^	0.005–0.010
Added sugar	28.1	23.2–35.7	32.8	^**^	26.7–40.2	21.3	^***^	14.3–30.1	6.2	4.8–7.6	6.8	^**^	5.3–8.4	4.4	^***^	3.1–5.9
Free sugar	29.9	24.1–36.5	33.6	^**^	27.7–43.1	23.1	^***^	14.9–32.4	6.6	4.9–7.8	6.9	^*^	5.5–9.0	4.9	^***^	3.3–6.4

Men (*n* = 92)																
Starch	204.4	187.1–226.8	203.6		171.7–233.6	200.5		137.2–172.1	34.4	31.3–38.4	34.7		30–41.2	36.3	^*^	29.3–42.6
Total sugar	66.4	51.6–77.2	65.9		54.6–81.7	58.1		49.1–65.2	11.0	8.7–13.4	11.4		9.0–14.3	10.6		8.3–12.9
Sucrose	30.6	21.6–37.8	38.0	^***^	28.9–43.5	28.3		21.7–32.8	5.2	3.6–6.4	6.6	^***^	4.7–7.6	5.0		3.6–6.4
Maltose	1.8	1.5–2.2	1.3	^***^	1.0–1.9	0.9	^***^	0.6–1.2	0.3	0.3–0.4	0.2	^***^	0.2–0.3	0.2	^***^	0.1–0.2
Lactose	5.0	3.1–9.2	4.3	^*^	2.3–8.4	5.4		4.8–8.3	0.8	0.5–1.6	0.8	^*^	0.4–1.4	1.0		0.4–1.5
Trehalose	0.18	0.12–0.25	0.12	^**^	0.09–0.19	0.12	^***^	0.08–0.17	0.03	0.02–0.04	0.02	^**^	0.01–0.03	0.02	^***^	0.02–0.04
Glucose	13.9	11.5–17.2	11.0	^***^	8.9–14.5	12.8	^**^	8.9–12.2	2.3	1.9–2.9	1.9	^***^	1.6–2.5	2.3	^*^	1.7–2.7
Fructose	11.3	8.8–13.6	9.5	^*^	7.0–13.2	10.5		8.2–12.5	1.9	1.5–2.3	1.6	^*^	1.2–2.3	1.8		1.4–2.5
Galactose	0.19	0.13–0.48	0.19		0.10–0.38	0.04	^***^	0.02–0.04	0.03	0.02–0.08	0.03		0.01–0.07	0.007	^***^	0.005–0.010
Added sugar	29.8	21.8–38.2	31.4	^*^	24.8–41	22.2	^***^	15.5–27.2	5.0	3.6–6.4	5.5	^*^	4.3–7.1	4.0	^***^	2.4–5.5
Free sugar	30.1	22–38.9	32.8	^*^	25.5–43.7	23.4	^***^	16.4–28.4	5.1	3.7–6.5	5.8	^*^	4.4–7.7	4.4	^**^	2.6–5.9

BDHQ1, first brief self-administered diet history questionnaire; DHQ1, first self-administered diet history questionnaire; DRs, dietary records; IQR, interquartile range.

^a^Energy adjustment was conducted according to the residual method or density method.

^b^Difference from 16-day DRs was investigated using Wilcoxon signed-rank test: ^*^P < 0.05, ^**^P < 0.01, ^***^P < 0.001.

The Spearman correlation coefficients between the 16-day DRs and DHQ1 were acceptable (≥0.31) for all carbohydrate variables (ranging from 0.43 for starch to 0.62 for lactose in women and from 0.31 for maltose to 0.67 for galactose in men), except for maltose (0.24) and trehalose (0.29) in women (Table 4). For the BDHQ1, the Spearman correlation coefficients were also acceptable for all variables (ranging from 0.32 glucose to 0.57 for added sugar in women and from 0.40 for added sugar to 0.64 for starch in men), except for maltose (0.17 in women and 0.26 in men) and galactose (−0.01 in women and 0.06 in men). The Spearman correlation coefficients for most sugars were higher in the DHQ1 than those in the BDHQ1 for both sexes. Similar findings were also observed with regard to the Spearman correlation coefficients between the 16-day DRs and the mDHQ and mBDHQ (eTable 2).

**Table 4.** Spearman correlation coefficient between intake of starch and 10 types of sugars estimated using the 16-day DRs and that estimated using the DHQ1 and BDHQ1 among Japanese women and men

	Women (*n* = 92)				Men (*n* = 92)			

	Residual model^a^		Density model^a^		Residual model^a^		Density model^a^	

	DHQ1	BDHQ1	DHQ1	BDHQ1	DHQ1	BDHQ1	DHQ1	BDHQ1
Starch	0.45	0.43	0.43	0.38	0.56	0.62	0.57	0.64
Total sugar	0.58	0.48	0.54	0.44	0.62	0.49	0.65	0.52
Sucrose	0.52	0.54	0.52	0.50	0.60	0.42	0.61	0.44
Maltose	0.23	0.17	0.24	0.17	0.33	0.23	0.31	0.26
Lactose	0.65	0.54	0.62	0.53	0.67	0.59	0.65	0.58
Trehalose	0.28	0.48	0.29	0.48	0.42	0.53	0.39	0.55
Glucose	0.47	0.34	0.45	0.32	0.58	0.44	0.60	0.48
Fructose	0.47	0.39	0.46	0.37	0.53	0.40	0.55	0.45
Galactose	0.61	−0.01	0.59	−0.01	0.61	0.06	0.67	0.06
Added sugar	0.54	0.53	0.53	0.57	0.51	0.42	0.54	0.40
Free sugar	0.54	0.51	0.53	0.55	0.52	0.41	0.56	0.41

BDHQ1, first brief self-administered diet history questionnaire; DHQ1, first self-administered diet history questionnaire; DRs, dietary records.

^a^Energy adjustment was conducted according to the residual method or density method.

**Figure 1.**
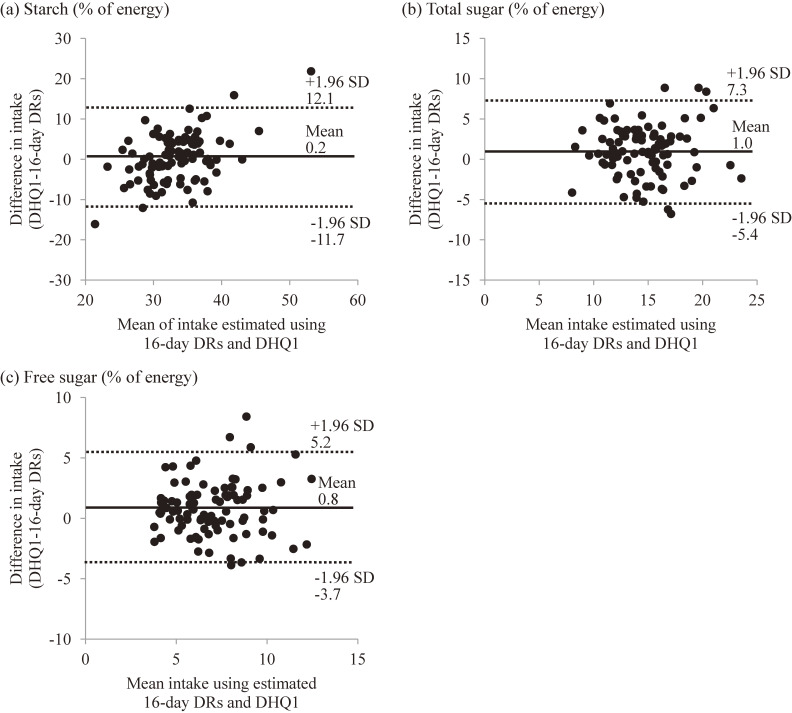
Brand-Altman plots between energy-adjusted intake (% of energy) of starch and total and free sugars estimated by 16-day DRs and that estimated by DHQ1 in 92 women; DRs, dietary records; DHQ1, first self-administered diet history questionnaire.

Figure 1 shows Brand-Altman plots for the intake of starch and total and free sugars in women. The limits of agreement appear to be wide (ranging from −11.7%E to 12.1%E for starch, from −5.4%E to 7.3%E for total sugar, and from −3.7%E to 5.2%E for free sugar), indicating poor agreement at the individual level. Similar results were obtained for the intake of other sugars in both sexes, irrespective of the dietary asse**s**sment questionnaires (Table 5 for the DHQ1 and BDHQ1 and eTable 3 for the mDHQ and mBDHQ).

**Table 5.** Brand-Altman statistics between intake of starch and 10 types of sugars estimated using the 16-day DRs and that estimated using the DHQ1 and BDHQ1 among Japanese women and men

	Residual model (g/d)^a^						Density model (% of energy)^a^					

	DHQ1			BDHQ1			DHQ1			BDHQ1		

	Mean difference^b^	Limits of agreement^c^		Mean difference^b^	Limits of agreement^c^		Mean difference^b^	Limits of agreement^c^		Mean difference^b^	Limits of agreement^c^	
Women (*n* = 92)												
Starch	1.5	−48.1,	51.2	1.0	−50.2,	52.2	0.2	−11.7,	12.1	2.7	−9.2,	14.5
Total sugar	6.2	−22.8,	35.2	−8.9	−40.1,	22.3	1.0	−5.4,	7.3	−1.1	−8.0,	5.8
Sucrose	7.3	−10.9,	25.6	−3.9	−21.9,	14.2	1.4	−2.5,	5.4	−0.4	−4.4,	3.6
Maltose	−0.5	−2.8,	1.7	−1.2	−3.1,	0.8	−0.1	−0.6,	0.3	−0.2	−0.6,	0.2
Lactose	1.2	−8.4,	10.9	−0.2	−7.9,	7.6	0.2	−1.8,	2.2	0.1	−1.7,	2.0
Trehalose	−0.02	−0.22,	0.18	−0.05	−0.21,	0.11	−0.006	−0.048,	0.036	−0.01	−0.04,	0.02
Glucose	−1.7	−9.3,	5.8	−2.3	−9.9,	5.3	−0.4	−2.0,	1.1	−0.4	−2.0,	1.3
Fructose	−0.2	−9.4,	9.1	−1.1	−9.6,	7.4	−0.1	−2.1,	1.9	−0.1	−2.1,	1.8
Galactose	0.02	−0.63,	0.67	−0.4	−1.1,	0.4	0.001	−0.132,	0.135	−0.08	−0.23,	0.08
Added sugar	4.0	−15.0,	22.9	−7.9	−29.6,	13.9	0.7	−3.5,	4.9	−1.4	−5.9,	3.1
Free sugar	4.4	−16.1,	24.8	−7.6	−30.2,	15.1	0.8	−3.7,	5.2	−1.4	−6.1,	3.3

Men (*n* = 92)												
Starch	−5.0	−79.4,	69.5	−5.2	−73.6,	63.1	0.9	−12.2,	13.9	2.0	−10.5,	14.5
Total sugar	1.7	−36.6,	40.0	−7.1	−44.9,	30.8	0.6	−5.3,	6.6	−0.5	−6.8,	5.8
Sucrose	6.4	−17.9,	30.6	−2.6	−25.1,	20.0	1.2	−2.6,	5.1	−0.1	−4.2,	3.9
Maltose	−0.5	−2.5,	1.6	−1.0	−2.8,	0.8	−0.07	−0.45,	0.30	−0.2	−0.5,	0.2
Lactose	−0.4	−8.7,	7.9	−0.3	−8.9,	8.2	0.01	−1.50,	1.52	0.04	−1.5,	1.6
Trehalose	−0.05	−0.26,	0.16	−0.06	−0.24,	0.12	−0.007	−0.043,	0.029	−0.008	−0.037,	0.022
Glucose	−2.6	−10.8,	5.6	−2.0	−11.9,	7.8	−0.4	−1.7,	1.0	−0.2	−1.8,	1.4
Fructose	−1.2	−10.5,	8.0	−0.8	−13.1,	11.6	−0.2	−1.7,	1.4	−0.005	−2.037,	2.027
Galactose	−0.04	−0.57,	0.49	−0.3	−0.9,	0.3	−0.005	−0.092,	0.082	−0.05	−0.15,	0.06
Added sugar	2.8	−22.6,	28.1	−7.2	−32.1,	17.7	0.6	−3.7,	5.0	−1.0	−5.5,	3.6
Free sugar	3.9	−22.5,	30.4	−5.9	−32.6,	20.7	0.8	−3.7,	5.3	−0.7	−5.6,	4.2

BDHQ1, first brief self-administered diet history questionnaire; DHQ1, first self-administered diet history questionnaire; DRs, dietary records.

^a^Energy adjustment was conducted according to the residual method or density method.

^b^Calculated as mean of differences obtained by subtracting the intake estimated using 16-day DRs from the intake estimated using the DHQ1 and BDHQ1.

^c^Calculated as mean difference ±1.96 standard deviations of mean difference.

Irrespective of dietary assessment method, >60% of starch intake was derived from rice in both sexes, whereas a variety of food groups were identified as major sources of total sugar, including fruits, confectionaries, sugars (as foods), vegetables, milk, sugar-sweetened beverages, and bread (Table 6). Compared with the 16-day DRs, both the DHQ1 and BDHQ1 generally identified the same food groups as major contributors to all other variables, with the following exceptions. For maltose, potatoes and mirin (Japanese traditional sweet seasoning made from rice, rice koji, and distilled alcohol) were found to be major sources (>30% of intake) only in the 16-day DRs (the latter was not assessed in DHQ or BDHQ). For galactose, yogurt and cheese were the top food sources in the 16-day DRs and the DHQ1 (>60% of intake) but not in the BDHQ1 (in which the food group was not assessed).

**Table 6.** Mean intake of starch and 10 types of sugars from each food group (g/d) estimated using the 16-day DRs, DHQ1, and BDHQ1 among Japanese women and men^a,b^

Nutrients	Food groups^c,d^	Women (*n* = 92)			Men (*n* = 92)		

		16-day DRs	DHQ1	BDHQ1	16-day DRs	DHQ1	BDHQ1
Starch	Total	155.1	156.6	27.2	206.7	201.8	201.5
	Rice	96.8	100.6	100.2	144.1	147.3	141.5
	Bread	16.4	21.3	14.3	17.2	21.3	15.3
	Noodles	16.1	14.2	10.7	20.2	16.5	16.3
	Others	25.9	20.6	30.9	25.2	16.7	28.5

Total sugar	Total	66.9	73.1	58.0	66.4	68.0	59.3
	Fruits	13.1	18.0	14.3	11.7	14.9	13.1
	Confectionaries	11.7	12.5	12.0	7.6	9.6	10.0
	Sugars	9.5	13.9	6.6	9.9	14.0	8.3
	Vegetables	8.5	5.9	8.8	9.0	5.2	8.8
	Milk	4.8	5.7	6.6	4.0	3.7	5.2
	Sugar sweetened beverages	3.7	3.2	2.5	6.8	5.3	4.1
	Bread	3.0	3.7	2.9	3.5	3.7	3.1
	Others	12.4	10.2	4.4	13.9	11.4	6.5

Sucrose	Total	32.2	39.5	28.3	31.0	37.3	28.4
	Confectionaries	10.4	10.5	10.8	6.7	8.0	8.8
	Sugars	8.6	13.4	6.5	8.9	13.5	8.2
	Fruits	5.3	7.1	5.7	4.9	6.1	5.3
	Vegetables	1.6	0.9	1.6	1.6	0.8	1.5
	Others	6.4	7.7	3.7	8.8	9.0	4.5

Maltose	Total	2.1	1.5	0.9	1.9	1.5	0.9
	Bread	0.5	0.6	0.3	0.6	0.6	0.4
	Potatoes	0.4	<0.1	<0.1	0.2	<0.1	<0.1
	Mirin^e,f^	0.4	NA	NA	0.4	NA	NA
	Confectionaries	0.3	0.6	0.3	0.2	0.5	0.3
	Noodles	0.3	0.1	0.1	0.3	0.1	0.1
	Others	0.2	0.3	0.1	0.3	0.3	0.1

Lactose	Total	7.4	8.7	7.3	6.3	5.9	6.0
	Milk	4.8	5.7	6.6	4.0	3.7	5.2
	Yogurt and cheese^e^	1.2	1.5	NA	0.9	1.0	NA
	Others	1.4	1.4	0.7	1.4	1.2	0.8

Trehalose	Total	0.17	0.16	0.12	0.20	0.15	0.14
	Mushrooms	0.12	0.11	0.10	0.13	0.10	0.10
	Bread	0.03	0.02	0.01	0.03	0.02	0.01
	Others	0.02	0.02	0.01	0.04	0.03	0.02

Glucose	Total	13.0	11.3	10.7	14.6	12.0	12.6
	Vegetables	3.6	2.6	3.9	3.9	2.3	3.9
	Fruits	3.2	4.4	3.6	2.6	3.7	3.3
	Miso	1.1	0.9	0.7	1.2	0.9	0.9
	Sugar sweetened beverages	0.9	0.9	0.9	1.4	1.4	1.4
	Mirin^e,f^	0.7	NA	NA	0.8	NA	NA
	Bread	0.6	0.3	0.3	0.7	0.3	0.3
	Other seasonings^e^	0.6	0.2	NA	0.7	0.1	NA
	Others	2.3	1.9	1.4	3.3	3.4	2.8

Fructose	Total	11.7	11.5	10.7	12.0	10.8	11.2
	Fruits	4.7	6.3	4.9	4.1	5.1	4.5
	Vegetables	3.3	2.4	3.4	3.5	2.1	3.4
	Sugar sweetened beverages	1.0	0.9	1.0	1.7	1.4	1.6
	Bread	0.9	0.5	0.4	0.9	0.5	0.4
	Other	1.9	1.4	1.0	1.9	1.7	1.3

Galactose	Total	0.39	0.41	0.03	0.33	0.29	0.04
	Yogurt and cheese^e^	0.29	0.35	NA	0.21	0.22	NA
	Soy sauce^e^	0.05	0.02	NA	0.07	0.03	NA
	Others	0.05	0.04	0.03	0.05	0.04	0.04

Added sugar	Total	30.3	34.3	22.4	31.1	33.9	23.9
	Confectionaries	10.9	11.2	11.5	7.1	8.7	9.4
	Sugars	9.5	13.8	6.6	9.9	13.9	8.3
	Sugar sweetened beverages	3.2	2.9	2.5	6.2	5.0	4.1
	Other seasonings^e^	1.2	0.5	NA	1.3	0.4	NA
	Others	5.6	5.9	1.9	6.7	5.9	2.0

Free sugars	Total	31.3	35.7	23.8	31.8	35.7	25.9
	Confectionaries	11.0	11.5	11.5	7.2	8.8	9.4
	Sugars	9.5	13.8	6.6	9.9	13.9	8.3
	Sugar sweetened beverages	3.3	3.0	2.5	6.2	5.1	4.1
	Other seasonings^e^	1.2	0.5	NA	1.3	0.4	NA
	Mirine^f^	1.1	NA	NA	1.3	NA	NA
	Others	5.3	7.0	3.2	6.0	7.5	4.0

BDHQ1, first brief self-administered diet history questionnaire; DHQ1, first self-administered diet history questionnaire; DRs, dietary records; NA, not assessed.

^a^Crude values are shown.

^b^Food groups shown are those providing 80% of cumulative contribution for the 16-day DRs in women.

^c^Twenty-eight food groups were defined based on the culinary usage and the similarity of nutrient profiles of the foods, mainly according to the Standard Tables of Food composition in Japan.^43^ They consisted of other grain products, pulses, nuts, seaweeds, fish and shellfish, meat, eggs, fat and oils, tea and coffee, fruit and vegetable juices, alcohol beverages, and salt in addition to the items listed in this table.

^d^Food groups are listed in descending order of the contribution for the 16-day DRs in women.

^e^Data not available because the respective food group was not assessed in the DHQ1 or BDHQ1.

^f^Japanese traditional sweet seasoning made from rice, rice koji and distilled alcohol.

## DISCUSSION

The present results showed a reasonable ranking ability of the DHQ and BDHQ for energy-adjusted intake of starch and of most sugars. Meanwhile, the median intake of most of these carbohydrates as estimated using the DHQ and BDHQ significantly differed from those estimated using the DRs, indicating that neither dietary assessment questionnaire was satisfactory for estimating the median intake. The results of Bland-Altman plots suggested that neither the DHQ nor the BDHQ was satisfactory for estimating the intake of starch and sugar subtypes at the individual level.

The Spearman correlation coefficients for the DHQ1 were acceptable (0.43–0.62 for women and 0.31–0.67 for men), except for maltose and trehalose in women, although the value for maltose was marginal (0.31) in men. For the BDHQ1, the correlation coefficients were acceptable (0.32–0.57 for women and 0.40–0.64 for men), except for maltose and galactose. Present findings are consistent with previous Western studies,^13^^–^^27^ where the median values of correlation coefficients for starch and sugar subtypes ranged from 0.33 (in Spain^19^) to 0.65 (in Australia^15^).

The correlation coefficients for starch and each of the sugars relied on the ability of the DHQ and BDHQ to estimate the intake of major food sources of these carbohydrate subtypes. For example, given that rice was the top contributor of starch in the present study, the relatively high correlations for rice intake in the DHQ1 and BDHQ1 (0.54–0.63 for women and 0.61 for men)^40^ were possible explanations for the moderate to relatively high correlations for starch (DHQ1: 0.43 for women and 0.57 for men and BDHQ1: 0.38 for women 0.64 for men). Similarly, the relatively acceptable correlations for food groups such as sugars (as food), confectionaries, vegetables, fruits, dairy products (including milk and other dairy products), and mushrooms as estimated using the DHQ1 and BDHQ1^40^ could lead to the acceptable correlations for sugar subtypes, such as total sugar, sucrose, added and free sugars, glucose, fructose, lactose, and trehalose (except for the result in DHQ1 for women).

Meanwhile, several food sources for maltose (eg, potatoes, mirin, breads, and confectionaries) might have contributed to the low correlations in the DHQ1 (0.24 for women and 0.31 for men) and BDHQ1 (0.17 for women and 0.26 for men). First, the correlations for potatoes were relatively low in the DHQ1 and BDHQ1 (0.13–0.17 for women and 0.21–0.30 for men); additionally, the increase in maltose in sweet potatoes (one of the components of potatoes) by cooking (ie, roasting and steaming) was not considered in these dietary assessment questionnaires. Second, mirin was not included in the DHQ and BDHQ. Moreover, for breads, the portion size and the proportion of components (ie, white breads and sweet buns with a sweet filling, such as azuki bean paste, custard cream, and jam) were fixed in the BDHQ, and thus the intake could be under- or overestimated relative to those in the DRs and DHQ, especially for white breads with a higher maltose content than that of sweet buns with a sweet filling.^30^^,^^31^ Furthermore, for caramels with a high maltose content (21.3 g/100 g of food),^30^^,^^31^ the fixed proportion to confectionaries were could be cause of under- or overestimation of the intake in the DHQ compared with those in the DRs. On the other hand, the correlations for galactose were low in the BDHQ1 (−0.01 for women and 0.06 for men) but not the DHQ1. One possible explanation is that the BDHQ did not assess yogurt and cheese, which were major food sources of galactose in the DRs and DHQ. Furthermore, the large number of food items, as well as the inclusion of the estimation of portion sizes, might have contributed to a more accurate assessment of intake in the DHQ, although this advantage would also be applicable for other sugars. Considering the low correlations for maltose (in the DHQ1 and BDHQ1) and galactose (only in the BDHQ1), we should interpret the intake of these sugars as estimated using each of the dietary assessment questionnaires with caution.

For the mDHQ and mBDHQ, similar results to those of the DHQ1 and BDHQ1 were observed. Ideally, dietary assessment should be conducted several times to capture habitual dietary intake over a long time period. That is, however, often difficult in many epidemiological studies. Therefore, the present finding suggested that the use of a single DHQ and BDHQ could be acceptable in epidemiological studies investigating the association of intake of starch and sugars and health issues over a long time period.

The present study has several limitations. First, the participants were not representative samples but volunteers who might be more health conscious than the general population. Thus, the results might not be directly applicable to the general Japanese population. Nevertheless, it should be noted that the mean height and weight of the participants were comparable to those of the general Japanese population.^49^ Additionally, the dietary assessment was conducted more than fifteen years ago (2002 to 2003). Therefore, dietary intake in current Japanese might differ from that in the present study. Second, although we used 16-day DRs as the gold standard, this method is based on a self-report, and the possibility of reporting error remains. However, reporting errors should be reduced through energy adjustment of the intake.^45^ Therefore, we included only energy-adjusted values in the present study. Third, the proportion of dietary intake from estimated (but not weighed) data was unknown because of a lack of information. Most of the dietary intake at home (127,491 food intakes; 77.5%) seems to be weighed while most of the dietary intake out of home (36,932 food intakes; 22.5%) seems to be estimated. Although the extent and direction was unknown, the dietary intake from estimated data was a possible cause of under- or overestimation of intakes of carbohydrate subtypes. However, this is a general limitation in dietary surveys. Finally, the median crude intake of maltose, trehalose, and galactose in 16-day DRs was low (median: <2.0 g/d) compared with other sugars (5.0–68.3 g/d) in this Japanese population as reported in a previous Japanese study.^30^ Thus, it might be challenging to obtain an accurate estimate of intake of these sugars based on a self-reported dietary assessment, including DRs. Therefore, caution is needed when interpreting the results of these three sugars compared with the results of other sugars.

In conclusion, this study indicated that the DHQ and BDHQ were not satisfactory in estimating the energy-adjusted intake of starch and sugar subtypes at the group or individual levels in Japanese adults. However, both the DHQ and BDHQ showed a reasonable ranking ability for the intake of starch, total sugars, sucrose, lactose, glucose, fructose, and added and free sugars, as well as maltose (only DHQ1 and mDHQ in men), trehalose (except for DHQ1 in women), and galactose (only DHQ1 and mDHQ in both sexes). The present findings support the potential use of these questionnaires for examining the association of starch and sugar intake with health status in epidemiological studies in Japan.

## APPENDIX A. SUPPLEMENTARY DATA

eTable 1. Intake of starch and 10 types of sugars estimated by the mDHQ and mBDHQ among Japanese women and men

eTable 2. Spearman correlation coefficient between intake of starch and 10 types of sugars estimated by the 16-day DRs and that estimated by the mDHQ and mBDHQ among Japanese women and men

eTable 3. Brand-Altman statistics between intake of starch and 10 types of sugars estimated by the 16-day DRs and that estimated by the mDHQ and mBDHQ among Japanese women and men
