# Supplementary material for: Relative Validity of Starch and Sugar Intake in Japanese Adults as Estimated With Comprehensive and Brief Self-Administered Diet History Questionnaires
Source: J Epidemiol. 2020 Aug 5;30(8):315–25. doi: 10.2188/jea.JE20190026 (PMC7348079; doi:10.2188/jea.JE20190026)
Supplement: Supplementary file 1 [file je-30-315-s001.pdf]

eTable 1. Intake of starch and 10 types of sugars estimated by the mDHQ and mBDHQ among Japanese women and men

	Residual model (g/d)[a]						Density model (% of energy)[a]					
	mDHQ[b]			mBDHQ[b]			mDHQ[b]			mBDHQ[b]		
	Median		IQR	Median		IQR	Median		IQR	Median		IQR
Women (n 92)												
Starch	152.7		141.3–167.2	150.3		138.2–165.4	33.0		30.1–36.9	35.4	***	32.6–39.3
Total sugar	68.3	*	60.6–79.5	51.2	***	44.3–60.9	14.7	*	12.5–17	12.2	**	10.5–14.3
Sucrose	37.4	***	30.9–43.2	24.8	***	20.5–31.6	8.0	***	6.7–9.3	5.8	*	4.7–7.4
Maltose	1.5	**	1.2–2.0	0.9	***	0.7–1.1	0.3	**	0.2–0.4	0.2	***	0.2–0.3
Lactose	8.2	***	5.2–10.7	6.9		5.1–8.9	1.75	***	1.2–2.3	1.6	*	1.2–2.1
Trehalose	0.13	**	0.09–0.17	0.10	***	0.07–0.14	0.03	***	0.02–0.04	0.02	***	0.02–0.03
Glucose	10.2	***	8.2–12.5	9.1	***	8.0–10.7	2.1	***	1.7–2.6	2.1	***	1.9–2.5
Fructose	9.9	***	8.2–12.8	9.1	***	7.4–10.9	2.0	**	1.6–2.8	2.1	**	1.7–2.5
Galactose	0.29		0.17–0.61	0.04	***	0.03–0.05	0.06		0.03–0.13	0.009	***	0.007–0.012
Added sugar	34.1	***	26.8–42.4	20.2	***	15.0–26.1	7.1	***	5.9–8.9	4.5	***	3.3–6.2
Free sugar	36.4	***	28.5–45.4	22.6	***	17.6–29.6	7.4	***	6.2–9.7	5.5	***	3.6–6.9
Men (n 92)												
Starch	198.9	*	173.6–223.1	193.6	**	173–222.6	34.0		29.9–39.6	35.3	**	31.4–40.6
Total sugar	67.5		56.0–83.8	59.2	***	46.4–67.4	11.7		9.5–14.1	10.6		8.5–12.2
Sucrose	38.6	***	30.4–44.3	27.2	**	19.8–33.6	6.4	***	4.9–7.6	4.8		3.5–6.1
Maltose	1.6		1.1–2.0	1.0	***	0.7–1.2	0.3		0.2–0.3	0.2	***	0.1–0.2
Lactose	5.8		3.5–8.9	6.6		3.2–9.0	1.0		0.6–1.6	1.2		0.6–1.6
Trehalose	0.13	***	0.10–0.17	0.11	***	0.08–0.16	0.02	***	0.02–0.03	0.02	***	0.02–0.03
Glucose	11.6	***	9.4–14	11.6	***	9.3–13.6	2.0	***	1.6–2.4	2.1	***	1.7–2.5
Fructose	10.1	**	7.6–13.1	10.0	*	7.8–12.9	1.7	*	1.3–2.2	1.8		1.4–2.3
Galactose	0.20		0.10–0.46	0.05	***	0.03–0.06	0.03		0.02–0.08	0.008	***	0.006–0.011
Added sugar	37.1	***	27.1–47.8	24.9	**	16.8–33.0	6.4	***	4.6–8.1	4.4	**	2.8–5.9
Free sugar	38.8	***	30.4–50.6	28.6	*	18.0–37.1	6.8	***	5–8.6	4.9		3.3–6.6

DRs, dietary records; mDHQ, mean of four self-administered diet history questionnaires; mBDHQ, mean of four brief self-administered diet history questionnaires; IQR, interquartile range.

[a]Energy adjustment was conducted according to the residual method or density method

[b]Difference from 16-day DRs (values were shown in Table 3) was investigated using Wilcoxon signed-rank test: *P<0.05, **P<0.01, ***P<0.001

eTable 2. Spearman correlation coefficient between intake of starch and 10 types of sugars estimated by the 16-day DRs and that estimated by the mDHQ and mBDHQ among Japanese women and men

	Women (n 92)				Men (n 92)			
	Residual model[a]		Density model[a]		Residual model[a]		Density model[a]	
	mDHQ	mBDHQ	mDHQ	mBDHQ	mDHQ	mBDHQ	mDHQ	mBDHQ
Starch	0.57	0.55	0.49	0.48	0.67	0.69	0.69	0.67
Total sugar	0.67	0.54	0.63	0.52	0.62	0.55	0.64	0.58
Sucrose	0.61	0.53	0.61	0.53	0.64	0.53	0.66	0.54
Maltose	0.18	0.16	0.17	0.16	0.31	0.26	0.32	0.30
Lactose	0.75	0.68	0.72	0.68	0.74	0.69	0.74	0.69
Trehalose	0.48	0.55	0.50	0.59	0.58	0.55	0.58	0.57
Glucose	0.51	0.43	0.50	0.45	0.54	0.51	0.59	0.54
Fructose	0.50	0.43	0.49	0.46	0.55	0.51	0.60	0.51
Galactose	0.81	0.10	0.80	0.10	0.77	0.05	0.81	0.08
Added sugar	0.59	0.49	0.59	0.53	0.51	0.36	0.54	0.40
Free sugar	0.56	0.47	0.57	0.51	0.51	0.37	0.53	0.43

DRs, dietary records; mDHQ, mean of four self-administered diet history questionnaires; mBDHQ, mean of four brief self-administered diet history questionnaires.

[a]Energy adjustment was conducted according to the residual method or density method

eTable 3. Brand-Altman statistics between intake of starch and 10 types of sugars estimated by the 16-day DRs and that estimated by the mDHQ and mBDHQ among Japanese women and men

	Residual model (g/d)[a]				Density model (% of energy)[a]			
	mDHQ		mBDHQ		mDHQ		mBDHQ	
	Mean difference[b]	Limits of agreement[c]	Mean difference[b]	Limits of agreement[c]	Mean difference[b]	Limits of agreement[c]	Mean difference[b]	Limits of agreement[c]
Women (n=92)								
Starch	0.07	-39.5, 39.7	-2.7	-40.8, 35.5	0.5	-10.1, 11.1	3.0	-6.6, 12.6
Total sugar	3.6	-20.1, 27.3	-13.4	-41.4, 14.7	0.7	-4.7, 6.1	-1.7	-7.7, 4.3
Sucrose	6.0	-9.1, 21.1	-6.0	-23.4, 11.4	1.3	-2.0, 4.6	-0.7	-4.4, 3.0
Maltose	-0.5	-2.6, 1.7	-1.1	-3.1, 0.8	-0.1	-0.6, 0.4	-0.2	-0.6, 0.2
Lactose	1.4	-4.4, 7.2	-0.3	-5.7, 5.1	0.3	-1.0, 1.6	0.1	-1.0, 1.3
Trehalose	-0.04	-0.19, 0.11	-0.06	-0.21, 0.08	-0.008	-0.04, 0.02	-0.01	-0.23, 0.02
Glucose	-2.4	-9.6, 4.9	-3.4	-10.2, 3.3	-0.5	-2.1, 1.0	-0.5	-2.0, 0.9
Fructose	-1.0	-8.7, 6.7	-2.2	-9.2, 4.8	-0.3	-2.0, 1.5	-0.3	-1.9, 1.3
Galactose	0.03	-0.37, 0.42	-0.3	-1.1, 0.4	0.006	-0.07, 0.09	-0.07	-0.23, 0.08
Added sugar	5.1	-12.9, 23.1	-8.3	-30.1, 13.5	1.0	-2.9, 5.0	-1.4	-6.0, 3.2
Free sugar	6.5	-13.4, 26.3	-7.0	-29.7, 15.6	1.3	-3.0, 5.7	-1.0	-5.9, 3.8
Men (n=92)								
Starch	-5.9	-60.8, 49.0	-8.0	-59.8, 43.8	0.1	-9.5, 9.7	1.4	-7.8, 10.6
Total sugar	3.6	-29.3, 36.4	-8.1	-42.2, 26.0	0.6	-4.6, 5.9	-0.7	-6.3, 4.8
Sucrose	7.3	-12.8, 27.5	-3.4	-24.3, 17.4	1.2	-2.0, 4.5	-0.3	-3.8, 3.2
Maltose	-0.2	-2.4, 1.9	-1.0	-2.7, 0.8	-0.05	-0.42, 0.33	-0.2	-0.5, 0.2
Lactose	0.3	-5.2, 5.9	0.4	-6.3, 7.1	0.09	-0.87, 1.05	0.2	-1.0, 1.3
Trehalose	-0.06	-0.23, 0.12	-0.07	-0.25, 0.10	-0.01	-0.04, 0.02	-0.01	-0.04, 0.02
Glucose	-2.6	-10.5, 5.3	-2.6	-10.8, 5.6	-0.4	-1.7, 0.8	-0.3	-1.7, 1.1
Fructose	-1.2	-9.7, 7.3	-1.1	-10.3, 8.1	-0.2	-1.6, 1.2	-0.06	-1.65, 1.53
Galactose	-0.02	-0.31, 0.28	-0.3	-0.9, 0.3	-0.0008	-0.0519, 0.0503	-0.05	-0.15, 0.06
Added sugar	7.1	-18.7, 32.9	-5.5	-32.6, 21.6	1.2	-3.1, 5.4	-0.7	-5.2, 3.8
Free sugar	8.8	-17.7, 35.4	-3.2	-31.2, 24.9	1.5	-3.0, 5.9	-0.3	-5.0, 4.5

DRs, dietary records; mDHQ, mean of four self-administered diet history questionnaires; mBDHQ, mean of four brief self-administered diet history questionnaires.

[a]Energy adjustment was conducted according to the residual method or density method

[b]Calculated as mean of differences obtained by subtracting the intake estimated by 16-day DRs from the intake estimated by the mDHQ and mBDHQ

[c]Calculated as mean difference ±1.96 standard deviations of mean difference
